# Epidemiological profile among pulmonary and extrapulmonary tuberculosis patients in Laayoune, Morocco

**DOI:** 10.11604/pamj.2020.37.56.21111

**Published:** 2020-09-15

**Authors:** Rkia Eddabra, Mounsef Neffa

**Affiliations:** 1 Higher Institute of Nurses Professions and Health Techniques of Laayoune, Ministry of Health, Avenue Colonel Major Habbouha Oueld Laâbid, Madinat Al Wahda I, Laayoune, Morocco,; 2 Laboratory of Bioresources, Biotechnology, Ethnopharmacology and Health, Applied Biochemistry to the Valorization of Bioresources, Faculty of Sciences, Mohamed 1st University, Oujda, Morocco.

**Keywords:** Tuberculosis, epidemiological profile, extrapulmonary, pulmonary, risk factors

## Abstract

**Introduction:**

despite major strides in the prevention, diagnosis and management of tuberculosis, the disease continues to be one of the most pressing global health problems, particularly in developing countries. The purpose of the present study was to describe the epidemiological profile among tuberculosis patients in Laayoune, Morocco.

**Methods:**

a retrospective study was conducted among tuberculosis patients (having extrapulmonary tuberculosis and pulmonary tuberculosis), registered in the diagnosis of tuberculosis and respiratory diseases reference center of Laayoune, between January 2017 and May 2018. Demographic characteristics, clinical presentation of TB and apparent risk factors of the disease were obtained from the medical case records of all patients.

**Results:**

during the study period, a total of 211 patients (125 males and 86 females) with tuberculosis were enrolled. The majority of cases (93.40%) were newly diagnosed and the segment with the pulmonary tuberculosis was 63.50%. The highest disease burden was found in the ≥15 year age group (92.40%; p=0.022). Men were more frequently affected by pulmonary tuberculosis (70.90%), while extrapulmonary tuberculosis was more commonly detected in women (61%) (p<0.0001). The most common sites of extrapulmonary disease were lymphatic (32.47%), pleural (16.88%) and spinal tuberculosis (15.58%). HIV infection and smoking seem to be the most important risk factors that affect host defense against TB infection.

**Conclusion:**

the results obtained in tuberculosis patients in Laayoune, Morocco, showed that active tuberculosis was associated with risk factors such as sex, age and smoking.

## Introduction

Tuberculosis (TB) is one of the most infectious diseases and leading causes of mortality and morbidity throughout the world [1]. TB is an airborne disease caused by infection with *Mycobacterium tuberculosis* (MTB), which typically affects the lungs (pulmonary tuberculosis: PTB). However, it also may affect any other organ of the body, which is known as extrapulmonary tuberculosis (EPTB) [2]. Patients with active pulmonary TB are the main sources of infection. The majority of people infected with MTB are recognized as having an asymptomatic latent TB infection (LTBI) [3]. TB is complicated by the emergence of drug-resistant TB, human immunodeficiency virus infection (HIV), poverty and lack of health services, especially in low-income countries, which experience the highest number of TB infections [4]. In 2018, the World Health Organization (WHO) estimated that 10 million people developed TB, with 1.5 million individuals (1.2 million among HIV-negative individuals and 251,000 among HIV-positive individuals) dying from the disease [5]. TB affects people of both sexes in all age groups, but the highest burden is in men (aged ≥15 years), who accounted for 57% of all TB cases in 2018 [5]. By comparison, women accounted for 32% and children (aged <15 years) for 11% of all cases. Among all TB cases, 8.6% were people living with HIV (PLHIV) [5].

TB-related morbidity and mortality remain particularly high in low and middle-income countries [6]. Many factors, such as socio-demographic factors (e.g. age, sex and occupation), environmental factors (e.g. indoor air pollution), factors that impair the host´s defense (e.g. smoking status, malnutrition, alcohol consumption, HIV and diabetes mellitus), as well as treatment challenges including the rise in drug-resistant TB, play a key role in accentuating the progression from exposure to infection with tuberculosis [7]. Tuberculosis is endemic and is a major public health problem in Morocco. Despite significant efforts to improve TB prevention, treatment and control, the incidence of tuberculosis is very high, with nearly 150 new cases per 100,000 inhabitants yearly, representing about 36,000 new cases per year, with more than 1,000 deaths per year [5]. This high incidence requires greater awareness and improved TB control measures. The main purpose of this study was to describe the clinical-epidemiological profile of patients with pulmonary and extrapulmonary tuberculosis in Laayoune, Morocco.

## Methods

A retrospective analysis was carried out on the medical records of patients, who presented to the diagnosis of tuberculosis and respiratory diseases reference center of Laayoune from January 2017 to May 2018. These cases emanate from the military hospital and various health centers, public pulmonologists and general practitioners in Laayoune province. Data were transcribed from each TB patient's medical record, using a specifically designed checklist.

**Data collection:** the data gathered include patients´ sociodemographic characteristics (age, sex and marital status), risk factors for TB infection (history of TB infection, exposure to an individual with TB, tobacco smoking and other comorbidities, such as diabetes, end-stage renal disease and human immunodeficiency virus infection) and clinical type of TB (cases were categorized by the site of the disease).

**Statistical analysis:** descriptive statistical methods were used to generate frequencies for categorical variables. Differences across groups (PTB and EPTB) were evaluated using the Chi-square test. The prevalence of TB was calculated for each risk factor with the respective odds ratios (OR) and confidence intervals (95% CI), estimating the strength of association and identifying risk factors of TB occurrence. Analyses were performed with statistical software (IBM, SPSS statistics for Windows, version 25.0. Armonk, NY: IBM Corp.). A p-value of less than 0.05 was considered statistically significant.

**Ethics statement:** this was a retrospective analysis of de-identified medical records. Individual informed consent was not obtained. The health establishment network services in Laayoune, Morocco approved the use of these data and only health professionals had access to the information. In order to ensure the confidentiality of the information, the names or identification numbers of TB patients were not included in the checklist.

## Results

A total of 238 tuberculosis patients were registered in the diagnosis of tuberculosis and respiratory diseases reference center during the study period. Unfortunately, 27 cases were not included due to the incomplete recording of information. Therefore, the final analysis was limited to 211 medical records of tuberculosis patients. Among these, 197 (93.40%) were new cases of TB and 14 (6.60%) were retreatment cases (Table 1).

**Table 1:** characteristics of registered TB patients in Laayoune, Morocco

Variables		Total number of TB patients n (%)	Type of TB		OR^a^ (95% CI)	P-value
			PTB n (%)	EPTB n (%)		
Gender	Male	125 (59.20)	95 (70.90)	30 (39.00)	0.29 (0.098 - 0.54)	<0.0001
	Female	86 (40.80)	39 (29.10)	47 (61.00)	1	
Age group years	1-14	16 (7.60)	8 (60.00)	8 (10.40)	0.44 (0.11 - 1.74)	0.022
	15-34	100 (47.40)	73 (54.50)	27 (35.10)	0.22 (0.09 - 0.53)	
	≥35	95 (45.00)	53 (39.60)	42 (54.50)	1	
Marital status	Married	96 (45.50)	55 (41)	41 (53.20)	19.61 (1.75 - 219.5)	0.315
	Single	100 (47.40)	69 (51.50)	31 (40.30)	14.90 (1.57 - 244)	
	Divorces	10 (4.70)	6 (4.50)	4 (5.20)	15 (0.85 - 263)	
	Widowed	5 (2.40)	4 (3.00)	1 (1.30)	1	
Patient type	Outpatients	176 (83.40)	114 (85.10)	62 (80.50)	0.55 (0.12 - 1.40)	0.392
	Inpatients	35 (16.60)	20 (14.90)	15 (19.50)	1	
Smoking cigarette	Yes	57 (27.00)	47 (35.10)	10 (13.00)	0.34 (0.13 - 0.87)	0.001
	No	154 (73.00)	87 (64.90)	67 (87.00)	1	
HIV sero-status	Positive	9 (4.30)	7 (5.20)	2 (2.60)	0.23 (0.03 - 1.63)	0.034
	Negative	36 (17.10)	29 (21.60)	7 (9.10)	0.67 (0.23 - 1.95)	
	Unknown	166 (78.70)	98 (73.10)	68 (88.30)	1	
History of exposure to an individual with TB	Yes	38 (18.00)	29 (21.60)	9 (11.70)	0.31 (0.12 - 0.83)	0.070
	No	173 (82.00)	105 (78.40)	68 (88.30)	1	
Diabetes	Yes	16 (7.60)	13 (9.70)	3 (3.90)	0.09 (0.02 - 0.43)	0.125
	No	195 (92.40)	121 (90.30)	74 (96.10)	1	
End-stage renal disease	Yes	2 (0.90)	1 (0.70)	1 (1.30)	0.30 (0.01 - 6.17)	0.690
	No	209 (99.10)	133 (99.30)	76 (98.70)	1	
Previous TB infection	New patient	197 (93.40)	122 (91.00)	75 (97.40)	4.89 (0.81 - 29.39)	0.074
	Re-treatment	14 (6.60)	12 (9.00)	2 (2.60)	1	
	Total	211 (100)	134 (63.50%)	77 (36.50%)		

: the values of 1 in this column represent data used as a reference for logistic regression analysis

**Socio-demographic characteristics of tuberculosis patients:** out of 211 patients, 125 (59.20%) were males and 86 (40.80%) were females (Table 1). The male to female ratio was 1.45. Concerning their marital status, 47.40% were single and 45.50% were married. The age distribution was significantly different (p = 0.022) between the PTB and EPTB groups. The mean age of the patients was 39.4 ± 18.6 years. In total, 54.50% fell under the 15-34 years old category for PTB and 54.50% were in the ≥35 years old category for EPTB.

**Clinical manifestations:** pulmonary tuberculosis (PTB) was present in 134 (63.50%) patients, while 77 (36.50%) patients had extrapulmonary tuberculosis (EPTB) (Table 1). The prevalence of EPTB was higher among females than males as compared to PTB (p<0.0001); the male to female ratio was 0.64 (30/47) for EPTB patients and 2.43 (95/39) for PTB patients.

**Sites of EPTB:** of all patients with EPTB, the most common infections were lymphatic (n=25; 32.47%), pleural (n=13; 16.88%), spinal (n=12; 15.58%), meningeal (n=9; 11.68%) and peritoneal (n=6; 7.79). Twelve EPTB patients (15.58%) had TB at other sites, namely the genitourinary, laryngeal, anal fistulas and cutaneous sites.

**Risk factors:** as compared to EPTB patients, a significantly higher proportion of PTB patients were smokers (35.10% vs 13.00%) (odds ratio (OR) 0.34, 95% confidence interval (CI) 0.13-0.87, p = 0.001) (Table 1). HIV status was known for only 45/211 (21.32%) patients (36 were HIV seronegative and nine were HIV seropositive in both the PTB and EPTB groups) and 63.99% (135/211) of patients had not been offered an HIV test, while 1.42% (3/211) had refused the test and 13.27% (28/211) had missing information (Table 1). A history of exposure to an individual with TB (21.60% vs 11.70%; p=0.07), a personal history of diabetes (9.70% vs 3.90%; p=0.125) and a history of treatment for TB infection in the past (9.00% vs 2.60%; p=0.074) were higher among PTB patients compared to EPTB patients.

## Discussion

Tuberculosis (TB) is among today´s most serious global health concerns. In developing countries, major global health organizations see TB as a critical public health challenge and are heavily invested in reducing TB-related mortality and morbidity [5]. In the present study, TB (all forms of TB) was more often diagnosed in men (n=125; 59.20%) than women (p <0.0001), which is consistent with other studies [8-10]. However, the reason for this gender difference has been debated and questioned frequently [9]. Some studies believe that these differences are in part due to societal factors (such as differential access to care), risk behaviors and activities (men are more likely to work outside the home) may render men more susceptible to pulmonary TB than women [10]. Other studies suggest that these differences might also have a biological basis (sex steroid hormones, genetic makeup of the sex chromosomes and sex-specific metabolic features) [11,12]. The two types of clinical manifestations of tuberculosis (TB) are pulmonary TB (PTB) and extrapulmonary TB (EPTB). Although the role of gender in TB is not yet clearly defined [10-12], PTB was higher in men (70.90%) and EPTB was higher in women (61%) (Table 1). This finding is in agreement with the results found in other studies. A study performed by Özvaran *et al*. indicated that EPTB had been more commonly observed in females (26.8%) as compared to males (13.1%) in Istanbul [13].

A study from Afghanistan and western Pakistan showed a higher occurrence of EPTB among females and the authors suggest that there may be a genetic predisposition to EPTB among indigenous ethnic populations [14]. However, this is finding contradicts results found in the United States and in Saudi Arabia, where EPTB rates are relatively higher among male patients [15,16]. Extra-pulmonary tuberculosis (EPTB) remains an important clinical problem because of difficulties in its diagnosis by conventional bacteriological methods (used at the diagnosis of tuberculosis and respiratory diseases reference center of Laayoune) and in monitoring its treatment. The clinical presentation of EPTB is extremely variable due to the site of involvement and to the aggressiveness of the disease [17]. Our study revealed that lymphatic system nodes and pleura were the most common sites of EPTB, similar to what others have reported [18-20]. In terms of age, the most affected groups were aged between 15 and 59 years (Table 1). Several studies documented that TB affects adults in their most productive years and causes considerable financial losses to the individual and family [21,22]. However, all age groups are at risk [5]. According to the results found in this study, 7.20% of the cases were children under the age of 14 years. Further research is required to explore the reasons for a higher prevalence among males, productive age groups and the roles of age and gender in disease causation.

Exogenous (such as contact with an infected individual) and endogenous (such as HIV, diabetes, tobacco smoke) risk factors may contribute to the increased susceptibility of an individual to develop TB infection. Our study showed that the factors influencing the risk of TB were more frequent in male patients and this finding aligns with many studies [23,24]. Multiple studies have shown that smoking or exposure to environmental tobacco smoke increases the rates of TB infection in incidence and severity [6,25]. Our data showed that smoking was substantially more prevalent among males (n=55; 44%). These results are comparable to those of Feng *et al*. 2014 [26], which reported that smokers are more likely to be male. Also, as in other studies, smoking was an independent risk factor for TB infection and pulmonary TB disease [27].

Tuberculosis is one of the most common opportunistic infections in HIV-infected patients, which has contributed to the resurgence of the disease in many parts of the world, particularly in developing countries [28]. The risk of developing active TB is estimated to be 20 to 30 times greater in HIV-seropositive patients than in those who are HIV-seronegative [29]. Our study showed that of all (n=45) HIV screenings conducted, n=9 were notified HIV-seropositive (Table 1) and n=135 (63.99%) patients were not offered an HIV test. Until now, there is no policy or program implementing routine HIV testing among TB patients in Morocco. Our results showed that 6.60% of patients had previously been treated and such patients may contribute to the transmission of *Mycobacterium tuberculosis* and the high TB prevalence rate [30]. This study had some obvious limitations. The present study was record-based; therefore, only a limited number of factors were analyzed. Some information about risk factors associated with TB infection was not recorded in the surveillance data that we used for the current study, such as HIV. This study recommends expanding HIV screening of TB patients and timely initiation of antiretroviral therapy (ART) to improve individual prognosis, reduce HIV transmission and reduce the burden of HIV and TB [31]. Another limitation was the lack of information regarding social factors (patient income; employment status; literacy status) and lifestyle (alcohol consumption).

## Conclusion

In conclusion, the study shows male sex preponderance among PTB cases and female preponderance among EPTB cases. Risk factors play an important role in the pathogenesis of *Mycobacterium tuberculosis*infection. Thus, early and accurate diagnosis of TB is crucial for containing the spread of TB among patients living in endemic areas.

### What is known about this topic

- Even after decades of availability of free anti-tuberculosis medications treatment and implementation of mass bacillus Calmette-Guerin (BCG) vaccination at birth, tuberculosis (TB) continues to be a major public health problem in Morocco;
- EPTB receives less interest than pulmonary tuberculosis because of its low infectious potential.

### What this study adds

- A higher proportion of adult TB patients (≥15 year age group) constituted the majority of patients treated in the diagnosis of tuberculosis and respiratory diseases reference center of Laayoune;
- The majority of cases of tuberculosis occur among males, but a higher occurrence of EPTB was observed in females.
